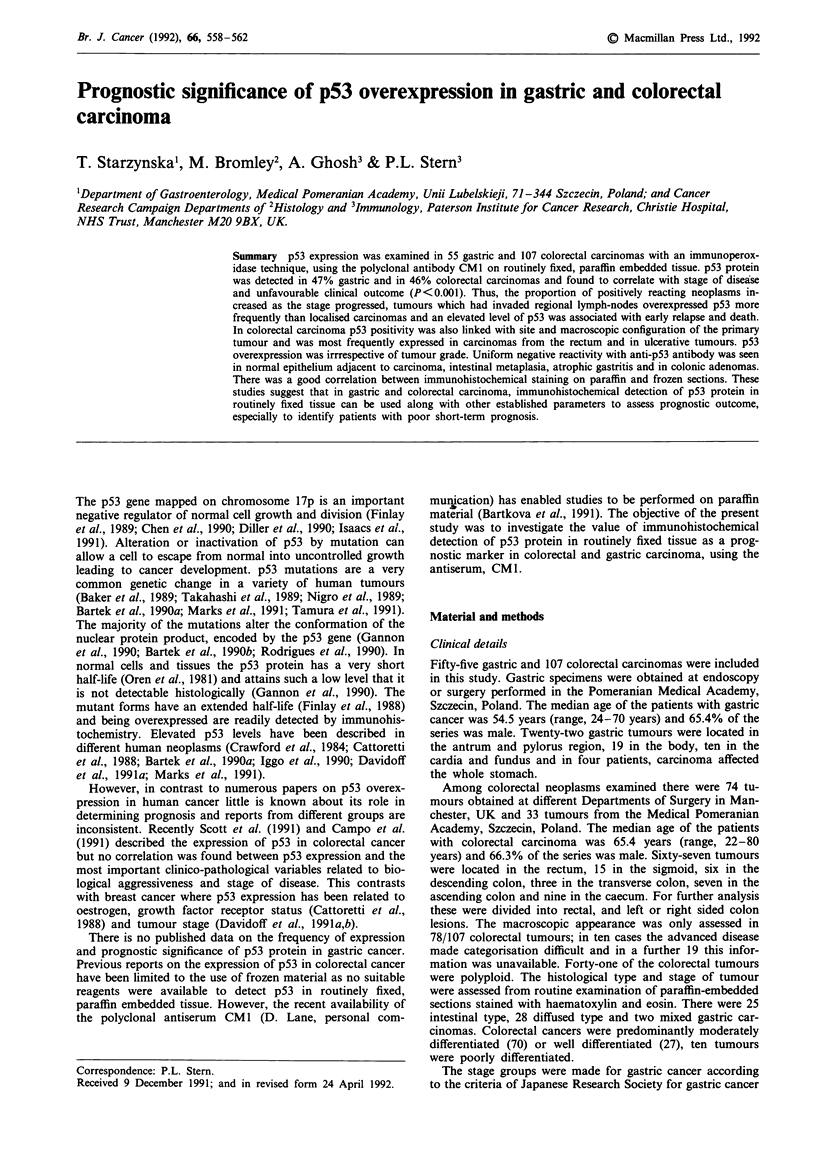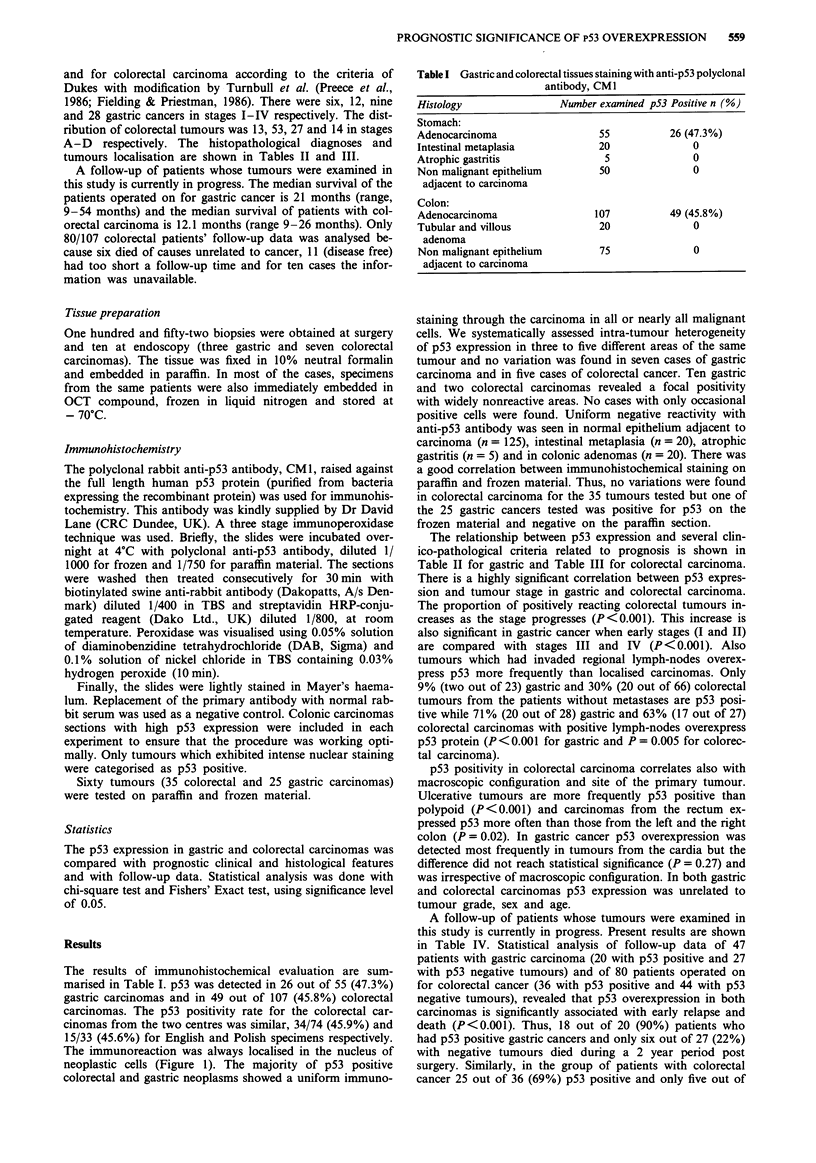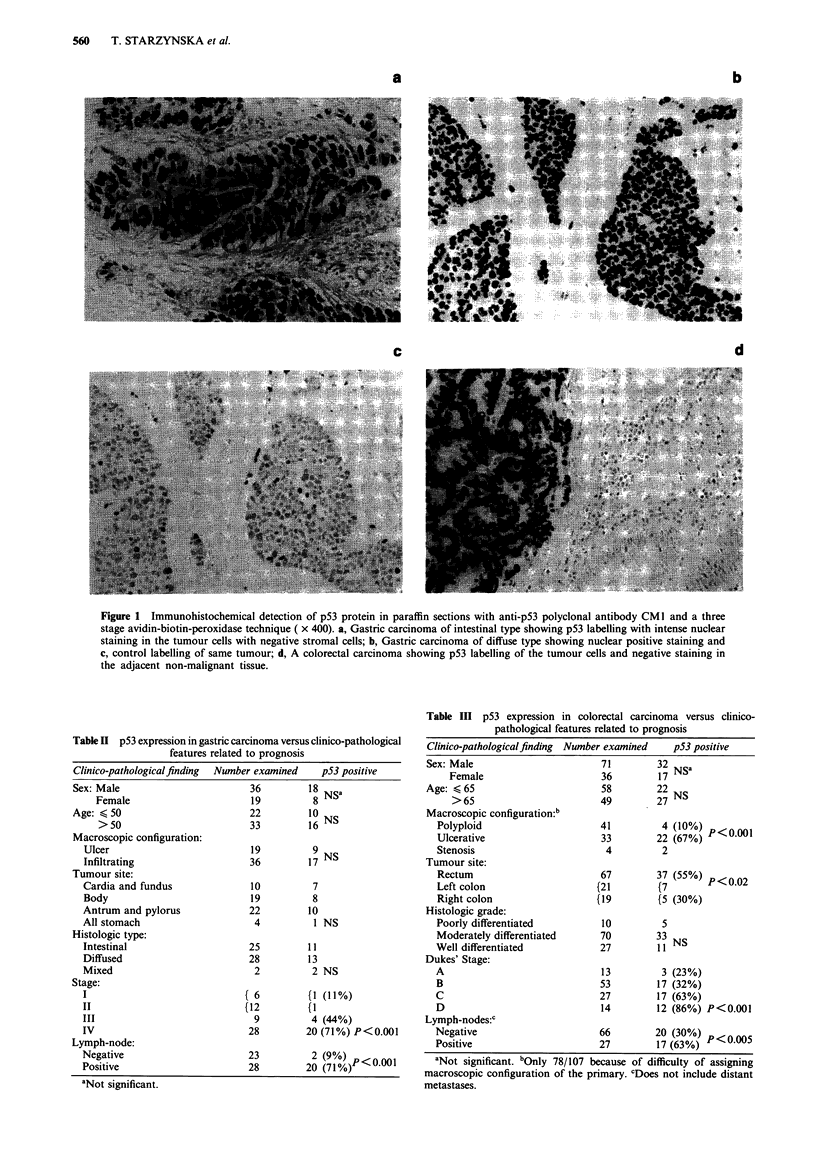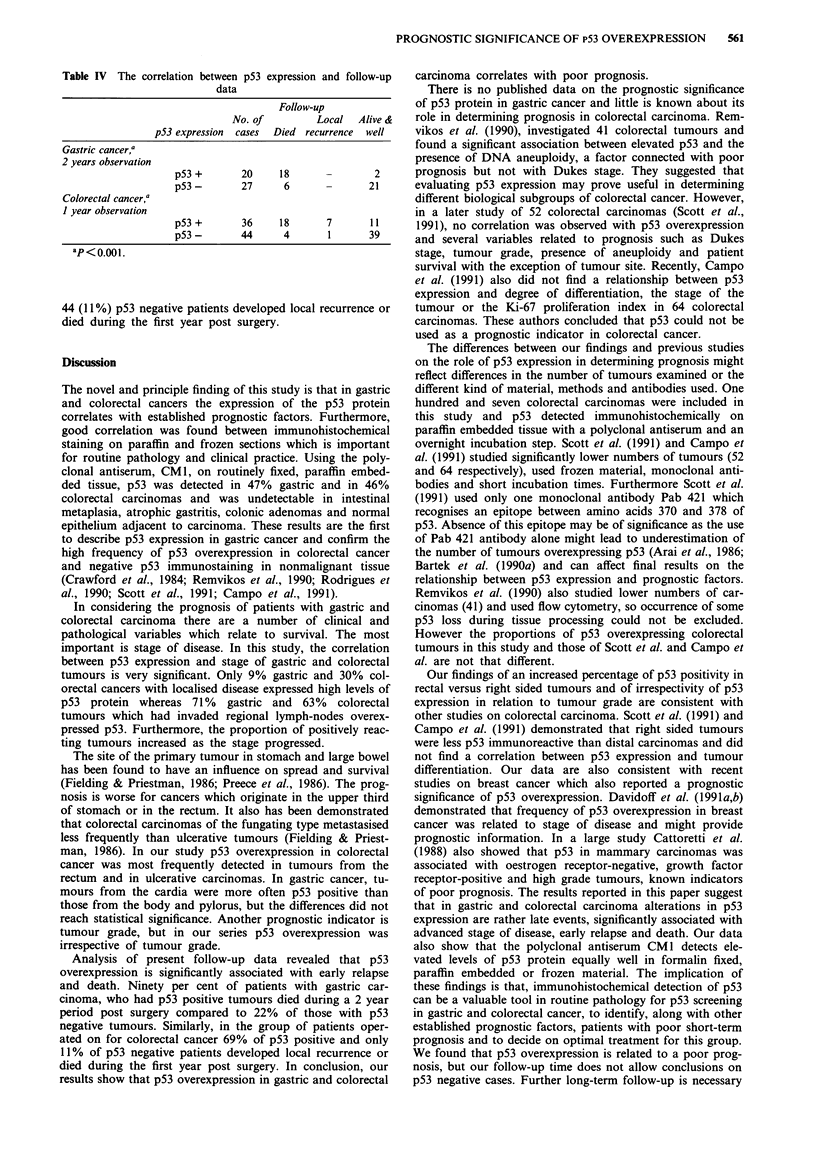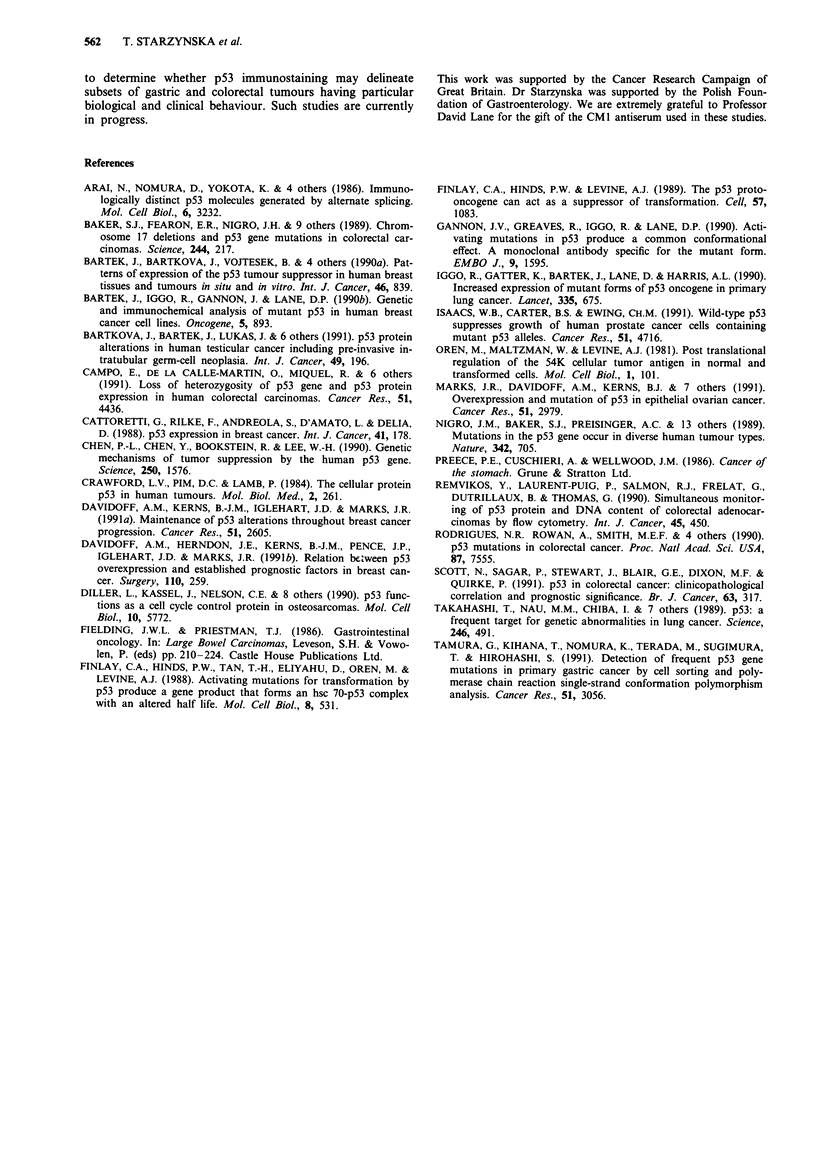# Prognostic significance of p53 overexpression in gastric and colorectal carcinoma.

**DOI:** 10.1038/bjc.1992.314

**Published:** 1992-09

**Authors:** T. Starzynska, M. Bromley, A. Ghosh, P. L. Stern

**Affiliations:** Department of Gastroenterology, Medical Pomeranian Academy, Szczecin, Poland.

## Abstract

**Images:**


Br. J. Cancer (1992), 66, 558-562 © Macmillan Press Ltd., 1992

### Prognostic significance of p53 overexpression in gastric and colorectal carcinoma

T. Starzynska[1], M. Bromley[2], A. Ghosh[3] & P.L. Stern[3]

[1]*Department of Gastroenterology, Medical Pomeranian Academy, Unii Lubelskieji, 71-344 Szczecin, Poland; and Cancer Research Campaign Departments of* [2]*Histology and* [3]*Immunology, Paterson Institute for Cancer Research, Christie Hospital, NHS Trust, Manchester M20 9BX, UK.*

**Summary** p53 expression was examined in 55 gastric and 107 colorectal carcinomas with an immunoperoxidase technique, using the polyclonal antibody CM1 on routinely fixed, paraffin embedded tissue. p53 protein was detected in 47% gastric and in 46% colorectal carcinomas and found to correlate with stage of disease and unfavourable clinical outcome ($P < 0.001$). Thus, the proportion of positively reacting neoplasms increased as the stage progressed, tumours which had invaded regional lymph-nodes overexpressed p53 more frequently than localised carcinomas and an elevated level of p53 was associated with early relapse and death. In colorectal carcinoma p53 positivity was also linked with site and macroscopic configuration of the primary tumour and was most frequently expressed in carcinomas from the rectum and in ulcerative tumours. p53 overexpression was irrespective of tumour grade. Uniform negative reactivity with anti-p53 antibody was seen in normal epithelium adjacent to carcinoma, intestinal metaplasia, atrophic gastritis and in colonic adenomas. There was a good correlation between immunohistochemical staining on paraffin and frozen sections. These studies suggest that in gastric and colorectal carcinoma, immunohistochemical detection of p53 protein in routinely fixed tissue can be used along with other established parameters to assess prognostic outcome, especially to identify patients with poor short-term prognosis.

The p53 gene mapped on chromosome 17p is an important negative regulator of normal cell growth and division (Finlay *et al.*, 1989; Chen *et al.*, 1990; Diller *et al.*, 1990; Isaacs *et al.*, 1991). Alteration or inactivation of p53 by mutation can allow a cell to escape from normal into uncontrolled growth leading to cancer development. p53 mutations are a very common genetic change in a variety of human tumours (Baker *et al.*, 1989; Takahashi *et al.*, 1989; Nigro *et al.*, 1989; Bartek *et al.*, 1990*a*; Marks *et al.*, 1991; Tamura *et al.*, 1991). The majority of the mutations alter the conformation of the nuclear protein product, encoded by the p53 gene (Gannon *et al.*, 1990; Bartek *et al.*, 1990*b*; Rodrigues *et al.*, 1990). In normal cells and tissues the p53 protein has a very short half-life (Oren *et al.*, 1981) and attains such a low level that it is not detectable histologically (Gannon *et al.*, 1990). The mutant forms have an extended half-life (Finlay *et al.*, 1988) and being overexpressed are readily detected by immunohistochemistry. Elevated p53 levels have been described in different human neoplasms (Crawford *et al.*, 1984; Cattoretti *et al.*, 1988; Bartek *et al.*, 1990*a*; Iggo *et al.*, 1990; Davidoff *et al.*, 1991*a*; Marks *et al.*, 1991).

However, in contrast to numerous papers on p53 overexpression in human cancer little is known about its role in determining prognosis and reports from different groups are inconsistent. Recently Scott *et al.* (1991) and Campo *et al.* (1991) described the expression of p53 in colorectal cancer but no correlation was found between p53 expression and the most important clinico-pathological variables related to biological aggressiveness and stage of disease. This contrasts with breast cancer where p53 expression has been related to oestrogen, growth factor receptor status (Cattoretti *et al.*, 1988) and tumour stage (Davidoff *et al.*, 1991*a,b*).

There is no published data on the frequency of expression and prognostic significance of p53 protein in gastric cancer. Previous reports on the expression of p53 in colorectal cancer have been limited to the use of frozen material as no suitable reagents were available to detect p53 in routinely fixed, paraffin embedded tissue. However, the recent availability of the polyclonal antiserum CM1 (D. Lane, personal communication) has enabled studies to be performed on paraffin material (Bartkova *et al.*, 1991). The objective of the present study was to investigate the value of immunohistochemical detection of p53 protein in routinely fixed tissue as a prognostic marker in colorectal and gastric carcinoma, using the antiserum, CM1.

Correspondence: P.L. Stern.
Received 9 December 1991; and in revised form 24 April 1992.

### Material and methods

### *Clinical details*

Fifty-five gastric and 107 colorectal carcinomas were included in this study. Gastric specimens were obtained at endoscopy or surgery performed in the Pomeranian Medical Academy, Szczecin, Poland. The median age of the patients with gastric cancer was 54.5 years (range, 24-70 years) and 65.4% of the series was male. Twenty-two gastric tumours were located in the antrum and pylorus region, 19 in the body, ten in the cardia and fundus and in four patients, carcinoma affected the whole stomach.

Among colorectal neoplasms examined there were 74 tumours obtained at different Departments of Surgery in Manchester, UK and 33 tumours from the Medical Pomeranian Academy, Szczecin, Poland. The median age of the patients with colorectal carcinoma was 65.4 years (range, 22-80 years) and 66.3% of the series was male. Sixty-seven tumours were located in the rectum, 15 in the sigmoid, six in the descending colon, three in the transverse colon, seven in the ascending colon and nine in the caecum. For further analysis these were divided into rectal, and left or right sided colon lesions. The macroscopic appearance was only assessed in 78/107 colorectal tumours; in ten cases the advanced disease made categorisation difficult and in a further 19 this information was unavailable. Forty-one of the colorectal tumours were polyploid. The histological type and stage of tumour were assessed from routine examination of paraffin-embedded sections stained with haematoxylin and eosin. There were 25 intestinal type, 28 diffused type and two mixed gastric carcinomas. Colorectal cancers were predominantly moderately differentiated (70) or well differentiated (27), ten tumours were poorly differentiated.

The stage groups were made for gastric cancer according to the criteria of Japanese Research Society for gastric cancer and for colorectal carcinoma according to the criteria of Dukes with modification by Turnbull *et al.* (Preece *et al.*, 1986; Fielding & Priestman, 1986). There were six, 12, nine and 28 gastric cancers in stages I–IV respectively. The distribution of colorectal tumours was 13, 53, 27 and 14 in stages A–D respectively. The histopathological diagnoses and tumours localisation are shown in Tables II and III.

A follow-up of patients whose tumours were examined in this study is currently in progress. The median survival of the patients operated on for gastric cancer is 21 months (range, 9–54 months) and the median survival of patients with colorectal carcinoma is 12.1 months (range 9–26 months). Only 80/107 colorectal patients' follow-up data was analysed because six died of causes unrelated to cancer, 11 (disease free) had too short a follow-up time and for ten cases the information was unavailable.

*Tissue preparation*

One hundred and fifty-two biopsies were obtained at surgery and ten at endoscopy (three gastric and seven colorectal carcinomas). The tissue was fixed in 10% neutral formalin and embedded in paraffin. In most of the cases, specimens from the same patients were also immediately embedded in OCT compound, frozen in liquid nitrogen and stored at − 70°C.

*Immunohistochemistry*

The polyclonal rabbit anti-p53 antibody, CM1, raised against the full length human p53 protein (purified from bacteria expressing the recombinant protein) was used for immunohistochemistry. This antibody was kindly supplied by Dr David Lane (CRC Dundee, UK). A three stage immunoperoxidase technique was used. Briefly, the slides were incubated overnight at 4°C with polyclonal anti-p53 antibody, diluted 1/1000 for frozen and 1/750 for paraffin material. The sections were washed then treated consecutively for 30 min with biotinylated swine anti-rabbit antibody (Dakopatts, A/s Denmark) diluted 1/400 in TBS and streptavidin HRP-conjugated reagent (Dako Ltd., UK) diluted 1/800, at room temperature. Peroxidase was visualised using 0.05% solution of diaminobenzidine tetrahydrochloride (DAB, Sigma) and 0.1% solution of nickel chloride in TBS containing 0.03% hydrogen peroxide (10 min).

Finally, the slides were lightly stained in Mayer's haemalum. Replacement of the primary antibody with normal rabbit serum was used as a negative control. Colonic carcinomas sections with high p53 expression were included in each experiment to ensure that the procedure was working optimally. Only tumours which exhibited intense nuclear staining were categorised as p53 positive.

Sixty tumours (35 colorectal and 25 gastric carcinomas) were tested on paraffin and frozen material.

*Statistics*

The p53 expression in gastric and colorectal carcinomas was compared with prognostic clinical and histological features and with follow-up data. Statistical analysis was done with chi-square test and Fishers' Exact test, using significance level of 0.05.

### Results

The results of immunohistochemical evaluation are summarised in Table I. p53 was detected in 26 out of 55 (47.3%) gastric carcinomas and in 49 out of 107 (45.8%) colorectal carcinomas. The p53 positivity rate for the colorectal carcinomas from the two centres was similar, 34/74 (45.9%) and 15/33 (45.6%) for English and Polish specimens respectively. The immunoreaction was always localised in the nucleus of neoplastic cells (Figure 1). The majority of p53 positive colorectal and gastric neoplasms showed a uniform immunostaining through the carcinoma in all or nearly all malignant cells. We systematically assessed intra-tumour heterogeneity of p53 expression in three to five different areas of the same tumour and no variation was found in seven cases of gastric carcinoma and in five cases of colorectal cancer. Ten gastric and two colorectal carcinomas revealed a focal positivity with widely nonreactive areas. No cases with only occasional positive cells were found. Uniform negative reactivity with anti-p53 antibody was seen in normal epithelium adjacent to carcinoma ($n = 125$), intestinal metaplasia ($n = 20$), atrophic gastritis ($n = 5$) and in colonic adenomas ($n = 20$). There was a good correlation between immunohistochemical staining on paraffin and frozen material. Thus, no variations were found in colorectal carcinoma for the 35 tumours tested but one of the 25 gastric cancers tested was positive for p53 on the frozen material and negative on the paraffin section.

**Table I** Gastric and colorectal tissues staining with anti-p53 polyclonal antibody, CM1

| *Histology* | *Number examined* | *p53 Positive n (%)* |
|---|---|---|
| Stomach: | | |
| Adenocarcinoma | 55 | 26 (47.3%) |
| Intestinal metaplasia | 20 | 0 |
| Atrophic gastritis | 5 | 0 |
| Non malignant epithelium adjacent to carcinoma | 50 | 0 |
| Colon: | | |
| Adenocarcinoma | 107 | 49 (45.8%) |
| Tubular and villous adenoma | 20 | 0 |
| Non malignant epithelium adjacent to carcinoma | 75 | 0 |

The relationship between p53 expression and several clinico-pathological criteria related to prognosis is shown in Table II for gastric and Table III for colorectal carcinoma. There is a highly significant correlation between p53 expression and tumour stage in gastric and colorectal carcinoma. The proportion of positively reacting colorectal tumours increases as the stage progresses ($P < 0.001$). This increase is also significant in gastric cancer when early stages (I and II) are compared with stages III and IV ($P < 0.001$). Also tumours which had invaded regional lymph-nodes overexpress p53 more frequently than localised carcinomas. Only 9% (two out of 23) gastric and 30% (20 out of 66) colorectal tumours from the patients without metastases are p53 positive while 71% (20 out of 28) gastric and 63% (17 out of 27) colorectal carcinomas with positive lymph-nodes overexpress p53 protein ($P < 0.001$ for gastric and $P = 0.005$ for colorectal carcinoma).

p53 positivity in colorectal carcinoma correlates also with macroscopic configuration and site of the primary tumour. Ulcerative tumours are more frequently p53 positive than polypoid ($P < 0.001$) and carcinomas from the rectum expressed p53 more often than those from the left and the right colon ($P = 0.02$). In gastric cancer p53 overexpression was detected most frequently in tumours from the cardia but the difference did not reach statistical significance ($P = 0.27$) and was irrespective of macroscopic configuration. In both gastric and colorectal carcinomas p53 expression was unrelated to tumour grade, sex and age.

A follow-up of patients whose tumours were examined in this study is currently in progress. Present results are shown in Table IV. Statistical analysis of follow-up data of 47 patients with gastric carcinoma (20 with p53 positive and 27 with p53 negative tumours) and of 80 patients operated on for colorectal cancer (36 with p53 positive and 44 with p53 negative tumours), revealed that p53 overexpression in both carcinomas is significantly associated with early relapse and death ($P < 0.001$). Thus, 18 out of 20 (90%) patients who had p53 positive gastric cancers and only six out of 27 (22%) with negative tumours died during a 2 year period post surgery. Similarly, in the group of patients with colorectal cancer 25 out of 36 (69%) p53 positive and only five out of

**Figure 1** Immunohistochemical detection of p53 protein in paraffin sections with anti-p53 polyclonal antibody CM1 and a three stage avidin-biotin-peroxidase technique ( × 400). **a**, Gastric carcinoma of intestinal type showing p53 labelling with intense nuclear staining in the tumour cells with negative stromal cells; **b**, Gastric carcinoma of diffuse type showing nuclear positive staining and **c**, control labelling of same tumour; **d**, A colorectal carcinoma showing p53 labelling of the tumour cells and negative staining in the adjacent non-malignant tissue.

**Table II** p53 expression in gastric carcinoma versus clinico-pathological features related to prognosis

| *Clinico-pathological finding* | *Number examined* | *p53 positive* |
|---|---|---|
| Sex: Male | 36 | 18 NS[a] |
| Female | 19 | 8 |
| Age: ⩽ 50 | 22 | 10 NS |
| > 50 | 33 | 16 |
| Macroscopic configuration: | | |
| Ulcer | 19 | 9 NS |
| Infiltrating | 36 | 17 |
| Tumour site: | | |
| Cardia and fundus | 10 | 7 |
| Body | 19 | 8 |
| Antrum and pylorus | 22 | 10 |
| All stomach | 4 | 1 NS |
| Histologic type: | | |
| Intestinal | 25 | 11 |
| Diffused | 28 | 13 |
| Mixed | 2 | 2 NS |
| Stage: | | |
| I | { 6 | {1 (11%) |
| II | {12 | {1 |
| III | 9 | 4 (44%) |
| IV | 28 | 20 (71%) $P<0.001$ |
| Lymph-node: | | |
| Negative | 23 | 2 (9%) $P<0.001$ |
| Positive | 28 | 20 (71%) |

[a]Not significant.

**Table III** p53 expression in colorectal carcinoma versus clinico-pathological features related to prognosis

| *Clinico-pathological finding* | *Number examined* | *p53 positive* |
|---|---|---|
| Sex: Male | 71 | 32 NS[a] |
| Female | 36 | 17 |
| Age: ⩽ 65 | 58 | 22 NS |
| > 65 | 49 | 27 |
| Macroscopic configuration:[b] | | |
| Polyploid | 41 | 4 (10%) $P<0.001$ |
| Ulcerative | 33 | 22 (67%) |
| Stenosis | 4 | 2 |
| Tumour site: | | |
| Rectum | 67 | 37 (55%) $P<0.02$ |
| Left colon | {21 | {7 |
| Right colon | {19 | {5 (30%) |
| Histologic grade: | | |
| Poorly differentiated | 10 | 5 |
| Moderately differentiated | 70 | 33 NS |
| Well differentiated | 27 | 11 |
| Dukes' Stage: | | |
| A | 13 | 3 (23%) |
| B | 53 | 17 (32%) |
| C | 27 | 17 (63%) |
| D | 14 | 12 (86%) $P<0.001$ |
| Lymph-nodes:[c] | | |
| Negative | 66 | 20 (30%) $P<0.005$ |
| Positive | 27 | 17 (63%) |

[a]Not significant. [b]Only 78/107 because of difficulty of assigning macroscopic configuration of the primary. [c]Does not include distant metastases.

**Table IV** The correlation between p53 expression and follow-up data

| | p53 expression | No. of cases | Follow-up Died | Local recurrence | Alive & well |
|---|---|---|---|---|---|
| *Gastric cancer,[a] 2 years observation* | | | | | |
| | p53 + | 20 | 18 | – | 2 |
| | p53 – | 27 | 6 | – | 21 |
| *Colorectal cancer,[a] 1 year observation* | | | | | |
| | p53 + | 36 | 18 | 7 | 11 |
| | p53 – | 44 | 4 | 1 | 39 |

[a]$P < 0.001$.

44 (11%) p53 negative patients developed local recurrence or died during the first year post surgery.

### Discussion

The novel and principle finding of this study is that in gastric and colorectal cancers the expression of the p53 protein correlates with established prognostic factors. Furthermore, good correlation was found between immunohistochemical staining on paraffin and frozen sections which is important for routine pathology and clinical practice. Using the polyclonal antiserum, CM1, on routinely fixed, paraffin embedded tissue, p53 was detected in 47% gastric and in 46% colorectal carcinomas and was undetectable in intestinal metaplasia, atrophic gastritis, colonic adenomas and normal epithelium adjacent to carcinoma. These results are the first to describe p53 expression in gastric cancer and confirm the high frequency of p53 overexpression in colorectal cancer and negative p53 immunostaining in nonmalignant tissue (Crawford *et al.*, 1984; Remvikos *et al.*, 1990; Rodrigues *et al.*, 1990; Scott *et al.*, 1991; Campo *et al.*, 1991).

In considering the prognosis of patients with gastric and colorectal carcinoma there are a number of clinical and pathological variables which relate to survival. The most important is stage of disease. In this study, the correlation between p53 expression and stage of gastric and colorectal tumours is very significant. Only 9% gastric and 30% colorectal cancers with localised disease expressed high levels of p53 protein whereas 71% gastric and 63% colorectal tumours which had invaded regional lymph-nodes overexpressed p53. Furthermore, the proportion of positively reacting tumours increased as the stage progressed.

The site of the primary tumour in stomach and large bowel has been found to have an influence on spread and survival (Fielding & Priestman, 1986; Preece *et al.*, 1986). The prognosis is worse for cancers which originate in the upper third of stomach or in the rectum. It also has been demonstrated that colorectal carcinomas of the fungating type metastasised less frequently than ulcerative tumours (Fielding & Priestman, 1986). In our study p53 overexpression in colorectal cancer was most frequently detected in tumours from the rectum and in ulcerative carcinomas. In gastric cancer, tumours from the cardia were more often p53 positive than those from the body and pylorus, but the differences did not reach statistical significance. Another prognostic indicator is tumour grade, but in our series p53 overexpression was irrespective of tumour grade.

Analysis of present follow-up data revealed that p53 overexpression is significantly associated with early relapse and death. Ninety per cent of patients with gastric carcinoma, who had p53 positive tumours died during a 2 year period post surgery compared to 22% of those with p53 negative tumours. Similarly, in the group of patients operated on for colorectal cancer 69% of p53 positive and only 11% of p53 negative patients developed local recurrence or died during the first year post surgery. In conclusion, our results show that p53 overexpression in gastric and colorectal carcinoma correlates with poor prognosis.

There is no published data on the prognostic significance of p53 protein in gastric cancer and little is known about its role in determining prognosis in colorectal carcinoma. Remvikos *et al.* (1990), investigated 41 colorectal tumours and found a significant association between elevated p53 and the presence of DNA aneuploidy, a factor connected with poor prognosis but not with Dukes stage. They suggested that evaluating p53 expression may prove useful in determining different biological subgroups of colorectal cancer. However, in a later study of 52 colorectal carcinomas (Scott *et al.*, 1991), no correlation was observed with p53 overexpression and several variables related to prognosis such as Dukes stage, tumour grade, presence of aneuploidy and patient survival with the exception of tumour site. Recently, Campo *et al.* (1991) also did not find a relationship between p53 expression and degree of differentiation, the stage of the tumour or the Ki-67 proliferation index in 64 colorectal carcinomas. These authors concluded that p53 could not be used as a prognostic indicator in colorectal cancer.

The differences between our findings and previous studies on the role of p53 expression in determining prognosis might reflect differences in the number of tumours examined or the different kind of material, methods and antibodies used. One hundred and seven colorectal carcinomas were included in this study and p53 detected immunohistochemically on paraffin embedded tissue with a polyclonal antiserum and an overnight incubation step. Scott *et al.* (1991) and Campo *et al.* (1991) studied significantly lower numbers of tumours (52 and 64 respectively), used frozen material, monoclonal antibodies and short incubation times. Furthermore Scott *et al.* (1991) used only one monoclonal antibody Pab 421 which recognises an epitope between amino acids 370 and 378 of p53. Absence of this epitope may be of significance as the use of Pab 421 antibody alone might lead to underestimation of the number of tumours overexpressing p53 (Arai *et al.*, 1986; Bartek *et al.* (1990*a*) and can affect final results on the relationship between p53 expression and prognostic factors. Remvikos *et al.* (1990) also studied lower numbers of carcinomas (41) and used flow cytometry, so occurrence of some p53 loss during tissue processing could not be excluded. However the proportions of p53 overexpressing colorectal tumours in this study and those of Scott *et al.* and Campo *et al.* are not that different.

Our findings of an increased percentage of p53 positivity in rectal versus right sided tumours and of irrespectivity of p53 expression in relation to tumour grade are consistent with other studies on colorectal carcinoma. Scott *et al.* (1991) and Campo *et al.* (1991) demonstrated that right sided tumours were less p53 immunoreactive than distal carcinomas and did not find a correlation between p53 expression and tumour differentiation. Our data are also consistent with recent studies on breast cancer which also reported a prognostic significance of p53 overexpression. Davidoff *et al.* (1991*a*,*b*) demonstrated that frequency of p53 overexpression in breast cancer was related to stage of disease and might provide prognostic information. In a large study Cattoretti *et al.* (1988) also showed that p53 in mammary carcinomas was associated with oestrogen receptor-negative, growth factor receptor-positive and high grade tumours, known indicators of poor prognosis. The results reported in this paper suggest that in gastric and colorectal carcinoma alterations in p53 expression are rather late events, significantly associated with advanced stage of disease, early relapse and death. Our data also show that the polyclonal antiserum CM1 detects elevated levels of p53 protein equally well in formalin fixed, paraffin embedded or frozen material. The implication of these findings is that, immunohistochemical detection of p53 can be a valuable tool in routine pathology for p53 screening in gastric and colorectal cancer, to identify, along with other established prognostic factors, patients with poor short-term prognosis and to decide on optimal treatment for this group. We found that p53 overexpression is related to a poor prognosis, but our follow-up time does not allow conclusions on p53 negative cases. Further long-term follow-up is necessary to determine whether p53 immunostaining may delineate subsets of gastric and colorectal tumours having particular biological and clinical behaviour. Such studies are currently in progress.

This work was supported by the Cancer Research Campaign of Great Britain. Dr Starzynska was supported by the Polish Foundation of Gastroenterology. We are extremely grateful to Professor David Lane for the gift of the CM1 antiserum used in these studies.

### References

ARAI, N., NOMURA, D., YOKOTA, K. & 4 others (1986). Immunologically distinct p53 molecules generated by alternate splicing. *Mol. Cell Biol.*, **6**, 3232.

BAKER, S.J., FEARON, E.R., NIGRO, J.H. & 9 others (1989). Chromosome 17 deletions and p53 gene mutations in colorectal carcinomas. *Science*, **244**, 217.

BARTEK, J., BARTKOVA, J., VOJTESEK, B. & 4 others (1990*a*). Patterns of expression of the p53 tumour suppressor in human breast tissues and tumours *in situ* and *in vitro*. *Int. J. Cancer*, **46**, 839.

BARTEK, J., IGGO, R., GANNON, J. & LANE, D.P. (1990*b*). Genetic and immunochemical analysis of mutant p53 in human breast cancer cell lines. *Oncogene*, **5**, 893.

BARTKOVA, J., BARTEK, J., LUKAS, J. & 6 others (1991). p53 protein alterations in human testicular cancer including pre-invasive intratubular germ-cell neoplasia. *Int. J. Cancer*, **49**, 196.

CAMPO, E., DE LA CALLE-MARTIN, O., MIQUEL, R. & 6 others (1991). Loss of heterozygosity of p53 gene and p53 protein expression in human colorectal carcinomas. *Cancer Res.*, **51**, 4436.

CATTORETTI, G., RILKE, F., ANDREOLA, S., D'AMATO, L. & DELIA, D. (1988). p53 expression in breast cancer. *Int. J. Cancer*, **41**, 178.

CHEN, P.-L., CHEN, Y., BOOKSTEIN, R. & LEE, W.-H. (1990). Genetic mechanisms of tumor suppression by the human p53 gene. *Science*, **250**, 1576.

CRAWFORD, L.V., PIM, D.C. & LAMB, P. (1984). The cellular protein p53 in human tumours. *Mol. Biol. Med.*, **2**, 261.

DAVIDOFF, A.M., KERNS, B.-J.M., IGLEHART, J.D. & MARKS, J.R. (1991*a*). Maintenance of p53 alterations throughout breast cancer progression. *Cancer Res.*, **51**, 2605.

DAVIDOFF, A.M., HERNDON, J.E., KERNS, B.-J.M., PENCE, J.P., IGLEHART, J.D. & MARKS, J.R. (1991*b*). Relation between p53 overexpression and established prognostic factors in breast cancer. *Surgery*, **110**, 259.

DILLER, L., KASSEL, J., NELSON, C.E. & 8 others (1990). p53 functions as a cell cycle control protein in osteosarcomas. *Mol. Cell Biol.*, **10**, 5772.

FIELDING, J.W.L. & PRIESTMAN, T.J. (1986). Gastrointestinal oncology. In: *Large Bowel Carcinomas*, Leveson, S.H. & Vowolen, P. (eds) pp. 210–224. Castle House Publications Ltd.

FINLAY, C.A., HINDS, P.W., TAN, T.-H., ELIYAHU, D., OREN, M. & LEVINE, A.J. (1988). Activating mutations for transformation by p53 produce a gene product that forms an hsc 70-p53 complex with an altered half life. *Mol. Cell Biol.*, **8**, 531.

FINLAY, C.A., HINDS, P.W. & LEVINE, A.J. (1989). The p53 proto-oncogene can act as a suppressor of transformation. *Cell*, **57**, 1083.

GANNON, J.V., GREAVES, R., IGGO, R. & LANE, D.P. (1990). Activating mutations in p53 produce a common conformational effect. A monoclonal antibody specific for the mutant form. *EMBO J.*, **9**, 1595.

IGGO, R., GATTER, K., BARTEK, J., LANE, D. & HARRIS, A.L. (1990). Increased expression of mutant forms of p53 oncogene in primary lung cancer. *Lancet*, **335**, 675.

ISAACS, W.B., CARTER, B.S. & EWING, CH.M. (1991). Wild-type p53 suppresses growth of human prostate cancer cells containing mutant p53 alleles. *Cancer Res.*, **51**, 4716.

OREN, M., MALTZMAN, W. & LEVINE, A.J. (1981). Post translational regulation of the 54K cellular tumor antigen in normal and transformed cells. *Mol. Cell Biol.*, **1**, 101.

MARKS, J.R., DAVIDOFF, A.M., KERNS, B.J. & 7 others (1991). Overexpression and mutation of p53 in epithelial ovarian cancer. *Cancer Res.*, **51**, 2979.

NIGRO, J.M., BAKER, S.J., PREISINGER, A.C. & 13 others (1989). Mutations in the p53 gene occur in diverse human tumour types. *Nature*, **342**, 705.

PREECE, P.E., CUSCHIERI, A. & WELLWOOD, J.M. (1986). *Cancer of the stomach*. Grune & Stratton Ltd.

REMVIKOS, Y., LAURENT-PUIG, P., SALMON, R.J., FRELAT, G., DUTRILLAUX, B. & THOMAS, G. (1990). Simultaneous monitoring of p53 protein and DNA content of colorectal adenocarcinomas by flow cytometry. *Int. J. Cancer*, **45**, 450.

RODRIGUES, N.R. ROWAN, A., SMITH, M.E.F. & 4 others (1990). p53 mutations in colorectal cancer. *Proc. Natl Acad. Sci. USA*, **87**, 7555.

SCOTT, N., SAGAR, P., STEWART, J., BLAIR, G.E., DIXON, M.F. & QUIRKE, P. (1991). p53 in colorectal cancer: clinicopathological correlation and prognostic significance. *Br. J. Cancer*, **63**, 317.

TAKAHASHI, T., NAU, M.M., CHIBA, I. & 7 others (1989). p53: a frequent target for genetic abnormalities in lung cancer. *Science*, **246**, 491.

TAMURA, G., KIHANA, T., NOMURA, K., TERADA, M., SUGIMURA, T. & HIROHASHI, S. (1991). Detection of frequent p53 gene mutations in primary gastric cancer by cell sorting and polymerase chain reaction single-strand conformation polymorphism analysis. *Cancer Res.*, **51**, 3056.